# Medical Complications After Aneurysmal Subarachnoid Hemorrhage: Analysis of Trends in US Admissions from 2006 to 2022

**DOI:** 10.1007/s12028-025-02443-6

**Published:** 2026-02-19

**Authors:** Ahmed Sabra, Karan Philip, Yaxel Levin-Carrion, Caryn J. Ha, Anurag Sahoo, Trong Huynh, David Landzberg, Amit Singla, James K. Liu, Kunakorn Atchaneeyasakuul, Priyank Khandelwal, Fadar Oliver Otite

**Affiliations:** 1 Department of Neurosurgery, Rutgers the State University of New Jersey, Newark, NJ USA; 2 Department of Neurology, University of Pittsburgh Medical Center, Pittsburgh, PA USA; 3 Department of Neurology, Rutgers the State University of New Jersey, Newark, NJ USA; 4 Department of Neurosurgery, Cooperman Barnabas Medical Center, RWJ Barnabas Health, Livingston, NJ USA; 5 Skull Base Institute of New Jersey, Neurosurgeons of New Jersey, NYU Langone Neurosurgery Network, Livingston, NJ USA; 6 Department of Neuroscience, Virginia Mason Medical Center, Seattle, WA USA

**Keywords:** Subarachnoid hemorrhage, Aneurysm, Ruptured, Cross-sectional studies, Hospitalization, Urinary tract infections, Pneumonia, Sepsis, Acute kidney injury (also indexed under Renal Insufficiency, Acute), Venous thrombosis, Pulmonary embolism, Gastrointestinal hemorrhage, Myocardial infarction

## Abstract

**Objectives:**

To analyze trends in the prevalence of medical complications in aneurysmal subarachnoid hemorrhage (aSAH) hospitalizations in the USA over the last decade.

**Methods:**

A serial cross-sectional study was performed using the 2006–2022 National Inpatient Sample. Adult (≥ 18 years) primary aSAH hospitalizations with and without complications were identified using International Classification of Diseases codes. Negative binomial regression models were used to evaluate the associations between complications, individualized hospitalization characteristics, and hospital outcomes.

**Results:**

Of 163,349 aSAH hospitalizations over the study period, 68.2% were female. The mean age was 55.6 years, and this increased over time (*p*-trend < 0.001). The mean National Inpatient Sample Subarachnoid Severity Score was 5.5 [standard error (SE) 0.06] and this also increased over time. Overall, 42.4% of hospitalizations had ≥ 1 medical complication. Urinary tract infections (UTI) (19.4%), pneumonia (15.4%), and sepsis (8.0%) were the most prevalent complications, while acute renal failure (ARF) (7.8%) was the most frequent noninfectious complication. The age- and sex-standardized prevalence of any medical complication remained stable over this study period, but there was marked heterogeneity in prevalence trends by complication type. ARF prevalence increased by nearly 300% [prevalence rate ratio (PRR): 1.04 per year, 95% confidence interval (CI): 1.02–1.04, *p* < 0.001] and deep venous thrombosis (DVT) prevalence increased by more than 200% (PRR:1.03, 95% CI: 1.01–1.04, *p* < 0.001) per year, while UTI and sepsis prevalence declined over this time (all *p*-trend < 0.001). Pneumonia prevalence declined in male only (*p*-trend < 0.05). Clipping was associated with higher DVT risk (PRR 1.24, 95% CI: 1.13–1.37) but lower gastrointestinal bleeding risk (PRR 1.14, 95% CI: 0.95–1.38) compared with coiling. All complications were significantly linked to poor outcomes (e.g., pneumonia: PRR 1.23, 95% CI: 1.20–1.30).

**Conclusions:**

The prevalence of infectious complications in aSAH has declined over the last decade, but this has been counterbalanced by a troubling increase in ARF and DVT prevalence. Given the strong association of all complications with poor outcomes, future studies focused on mitigating the prevalence of complications are needed to help improve aSAH outcomes.

**Supplementary Information:**

The online version contains supplementary material available at https://doi.org/10.1007/s12028-025-02443-6.

## Introduction

Aneurysmal subarachnoid hemorrhage (aSAH) is one of the most devastating forms of stroke, accounting for approximately 3–5% of all strokes [1, 2], but with a disproportionate stroke morbidity burden. Medical complications contribute significantly to aSAH-associated morbidity and mortality [1], but data on the overall burden of medical complications in aSAH hospitalizations in the USA are sparse. With improvement in general and neurocritical care of patients with aSAH over the last two decades [3, 4], a decrease in the prevalence of medical complications might be expected. However with the slow shift in the demographic and clinical profile of aSAH hospitalizations in the US toward elderly individuals^5^ with more preexisting comorbidities [5, 6], an increased burden of medical complications might be seen.

This study aims to quantify and evaluate trends in the prevalence of selected noninfectious [acute myocardial infarction (AMI), acute renal failure (ARF), deep venous thrombosis (DVT), pulmonary embolism (PE), and gastrointestinal bleeding (GIB)] and infectious [urinary tract infection (UTI), pneumonia, and sepsis] medical complications in various demographic subgroups of aSAH admissions in the USA.

## Methods

### Study Design and Data Source

This is a serial cross-sectional study conducted using the 2006–2022 Nationwide Inpatient Sample (NIS). The NIS is the largest all-payer, inpatient care database in the USA and comprises a 20% stratified random sample of all US hospital discharges. Sampling weights provided in the NIS allow for calculation of national estimates. Each individual hospital discharge in the NIS is de-identified, so all discharges were considered independent. Further details on the NIS design are available at https:/hcup-us.ahrq.gov.

### Study Population

All adult (≥ 18 years old) hospitalizations with a primary or secondary aSAH diagnosis were identified from the 2006–2022 NIS. International Classification of Diseases (ICD) Ninth Revision codes 430.xx were used for patients before October 2015 and ICD-10 codes I60.xx were used after this date. These codes are concordant with physician-diagnosed SAH in > 90% of cases [7]. We relied on methods previously validated by others [8] to identify the subset of aSAH admissions by restricting admissions to only those with codes for aneurysmal clipping or coiling using codes, as enumerated in eTable 1. Admissions with codes corresponding to head trauma or arteriovenous malformations were excluded.

### Outcomes and Covariates Definition

Most complications were ascertained using the Healthcare Cost and Utilization Project (HCUP)-defined constellation of ICD-9 and ICD-10 codes contained in the HCUP Clinical Classification Software (CCS) for ICD-9 (https://www.hcup-us.ahrq.gov/toolssoftware/ccs/AppendixASingleDX.txt) and ICD-10 https://www.hcup-us.ahrq.gov/toolssoftware/ccsr/ccsr_archive.jsp, as has been described previously [9]. Hospitalizations with DVT were ascertained using ICD-9 codes 453.4X, 453.8x, 451.11, 451.19, 451.2, and 451.9 or ICD-10 codes I82.4X, I82.X, I82.890, and I82.90, while PE was identified using code 415.1X, excluding 415.12 for septic pulmonary embolism and ICD-10 code I26.XX. In-hospital deaths were captured using the HCUP variable labeled “DIED.” We defined poor outcome using the dichotomous NIS Subarachnoid Hemorrhage Outcome Measure (NIS‐SOM), which has been shown in prior studies to have a strong concordance with modified Rankin Scale greater than 2 at discharge [10, 11]. The NIS‐SOM comprises in-hospital mortality; discharge to intermediate, long‐term care, or skilled nursing facility; or placement of tracheostomy or gastrostomy tube.

We evaluated the baseline comorbidity burden of aSAH admissions using the Elixhauser Comorbidity Index, a validated comorbidity measure consisting of 31 likely chronic conditions. Severity of aSAH was evaluated using the previously validated Nationwide Inpatient Sample Subarachnoid Hemorrhage (SAH) Severity Score [NISSSS] [10], using codes for aphasia, hydrocephalus, hydrocephalus requiring treatment, mechanical ventilation, cranial nerve palsy, coma, and stupor, as outlined in eTable 1.

### Statistical Analysis

Baseline characteristics of aSAH hospitalizations were evaluated using descriptive statistics. We used weights provided in the NIS (including NIS TRENDWT accounting for the NIS redesign in 2012), to compute the overall national weighted prevalence of each complication and in subgroups characterized by age and sex. Trends in the prevalence of complications over time were evaluated using negative binomial regression models with each complication as the dependent variable and year as a continuous independent variable, with significance in trend over time evaluated using the Wald test.

We further used a series of nested regression models to evaluate the association of each complication with selected demographic (age, sex, race) and clinical factors (NISSSS, Elixhauser Comorbidity Index, coiling vs. clipping). Fully adjusted models were adjusted for hospital level factors, including hospital region and location/teaching status, in addition to smoking status and insurance status. Similar models with NIS-SOM as the dependent variable were used to evaluate the association of complications with aSAH outcome.

A *p*-value threshold of < 0.05 was required for statistical significance. Because this study was descriptive and exploratory in nature, *p*-values should be interpreted cautiously. Given the very large sample size and the number of comparisons performed, small differences may reach statistical significance (*p* < 0.05) even when the absolute effect size is minimal and of uncertain clinical relevance. Accordingly, *p*-values are reported to allow the reader to assess statistical patterns, but they should not be viewed as definitive evidence of meaningful associations. No adjustments were made for multiple comparisons, consistent with methodological guidance for exploratory analyses [12, 13]. In interpreting the results, emphasis should be placed on effect sizes, prevalence ratios, and temporal trends rather than on statistical significance alone. All analyses were performed by FOO in Stata 16 (StataCorp LP, College Station, TX, USA). The complex NIS survey was taken into account in all analyses by application of relevant probability sampling weight as recommended to all analyses.

### Missing Data

Of all variables of interest, data on race/ethnicity were missing in 3.1%. Data on hospital location/teaching status were missing in 0.5% and on insurance status in 0.2%. Missing variables with respect to race were categorized into an “unknown/other” category, and for the other variables, they were categorized to the dominant category given the very small numbers.

### Ethics Statement

This study utilized de-identified, publicly available data from the Nationwide Inpatient Sample (NIS), and as such, was exempt from institutional review board (IRB) approval. Because the data are de-identified and do not contain any personally identifiable information, patient consent was not required or applicable.

## Results

### Baseline Characteristics

**Fig. 1:**
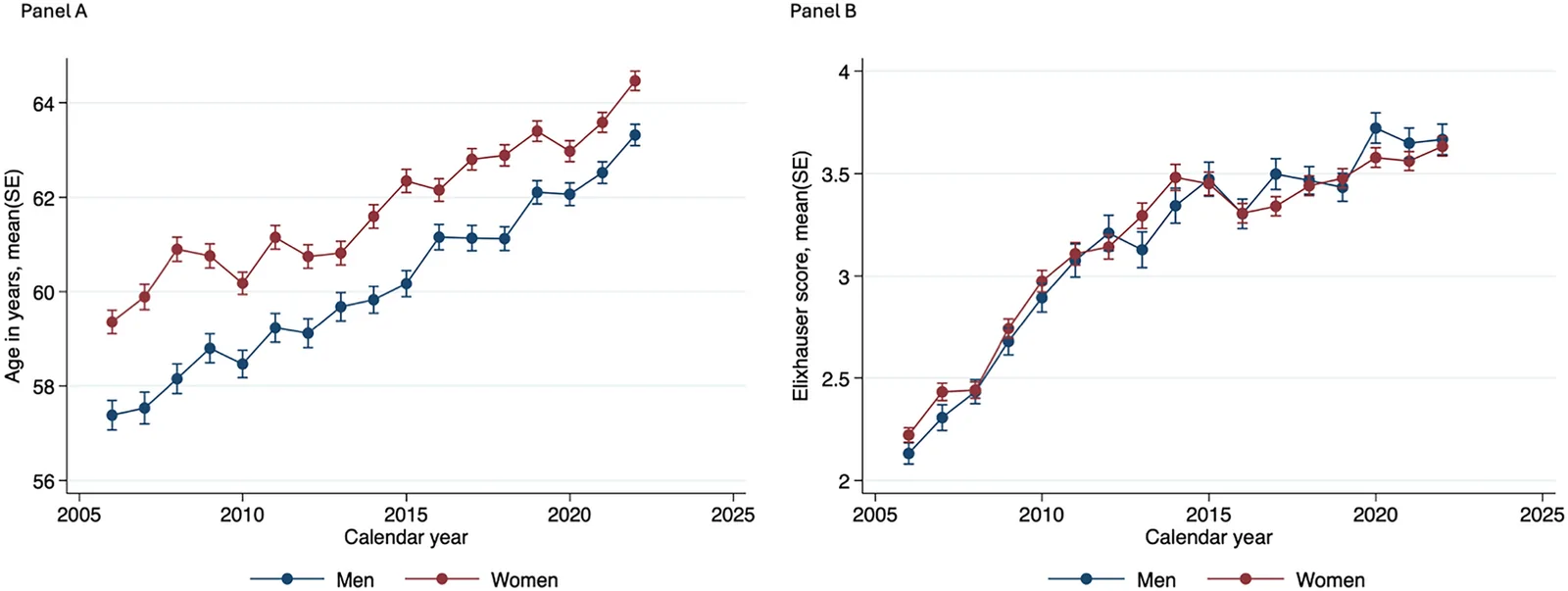
Trends in age and Elixhauser Comorbidity Score in aneurysmal subarachnoid hemorrhage in the USA from 2006 to 2022. Circles represent means and error bars represent standard errors of the mean

A total of 163,349 weighted hospitalizations for aSAH were included in our analysis. As presented in eTable 2, 68.2% of all hospitalizations were in female. The mean age of hospitalizations in female (56.8 years) was marginally higher than that of male (53.2 years) (*p* < 0.001), while the mean age of all admissions increased over time (*p*-for-trend < 0.001) (Fig. 1). The proportion of hospitalizations in ages 60–79 and ≥ 80 years increased over time just as the proportion in all age groups < 60 years declined over time (eFig. 1). The average number of comorbidities in all admissions as measured by the Elixhauser Comorbidity Score was 3.2 but there was almost a twofold increase in the number of baseline comorbidities across the study period (Fig. 1). Mean length of stay (LOS) was 18.8 days [standard error (SE): 0.13 days] (eFig. 2). The mean clinical severity of SAH (as measured by the NISSSS) was 5.5 (SE 0.06), and this also increased significantly over time in both sexes (*p*-for-trend < 0.001) (eFig. 3) Approximately two-thirds (65.5%) of all hospitalizations were for coiling, while one-third underwent aneurysm clipping.

### Overall Prevalence of Medical Complications

**Fig. 2:**
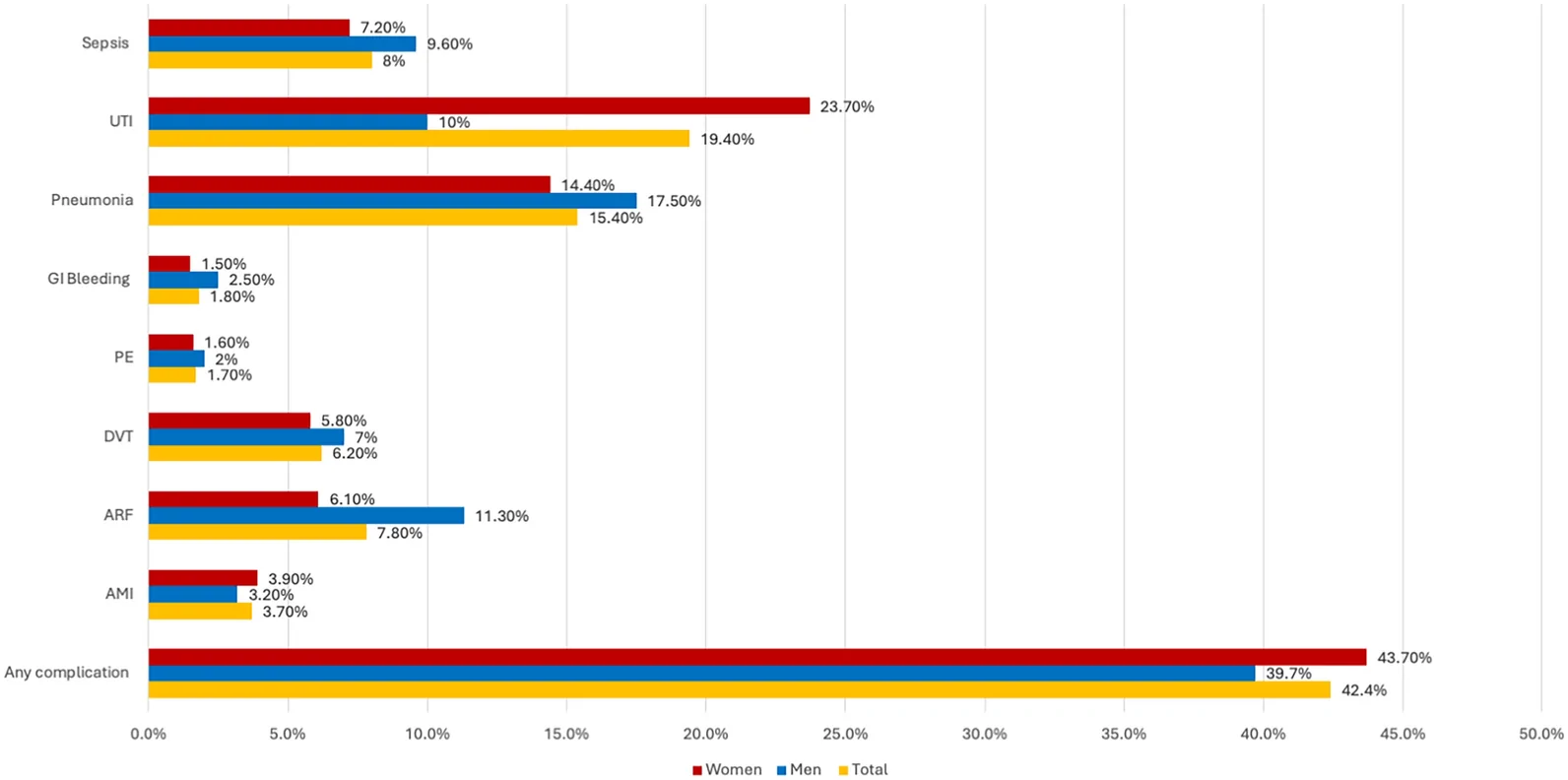
Age- and sex-standardized prevalence of medical complications in aneurysmal subarachnoid hemorrhage hospitalizations in the USA from 2006 to 2022. *p*-value for trend toward increasing prevalence with age (*p* < 0.05) for all comorbidities, except for PE in female

Across the study period, 42.4% of all aSAH hospitalizations had at least one medical complication, but overall complication burden differed by age and by sex. Overall, infectious complications, including UTI (19.4%), pneumonia (15.4%), and sepsis (8.0%), were the most prevalent complications, while AMI (3.7%), GIB (1.8%), and PE (1.7%) were the least prevalent. ARF (7.8%) was the most common noninfectious complication (Fig. 2).

Further stratification by age elucidated important heterogeneity in prevalence of complications. There was generally a significant trend toward increasing prevalence of most complications with advancing age (all *p*-for-trends < 0.05) except for pulmonary embolism in female (eFig. 4). Greater than 1 in 4 hospitalizations in male ≥ 80 years (26.8%) had comorbid pneumonia, while approximately 1 in 3 female ≥ 80 years (30.8%) had comorbid UTI (eFig. 4).

### Trends in Age- and Sex-Standardized Prevalence of Complications

**Fig. 3:**
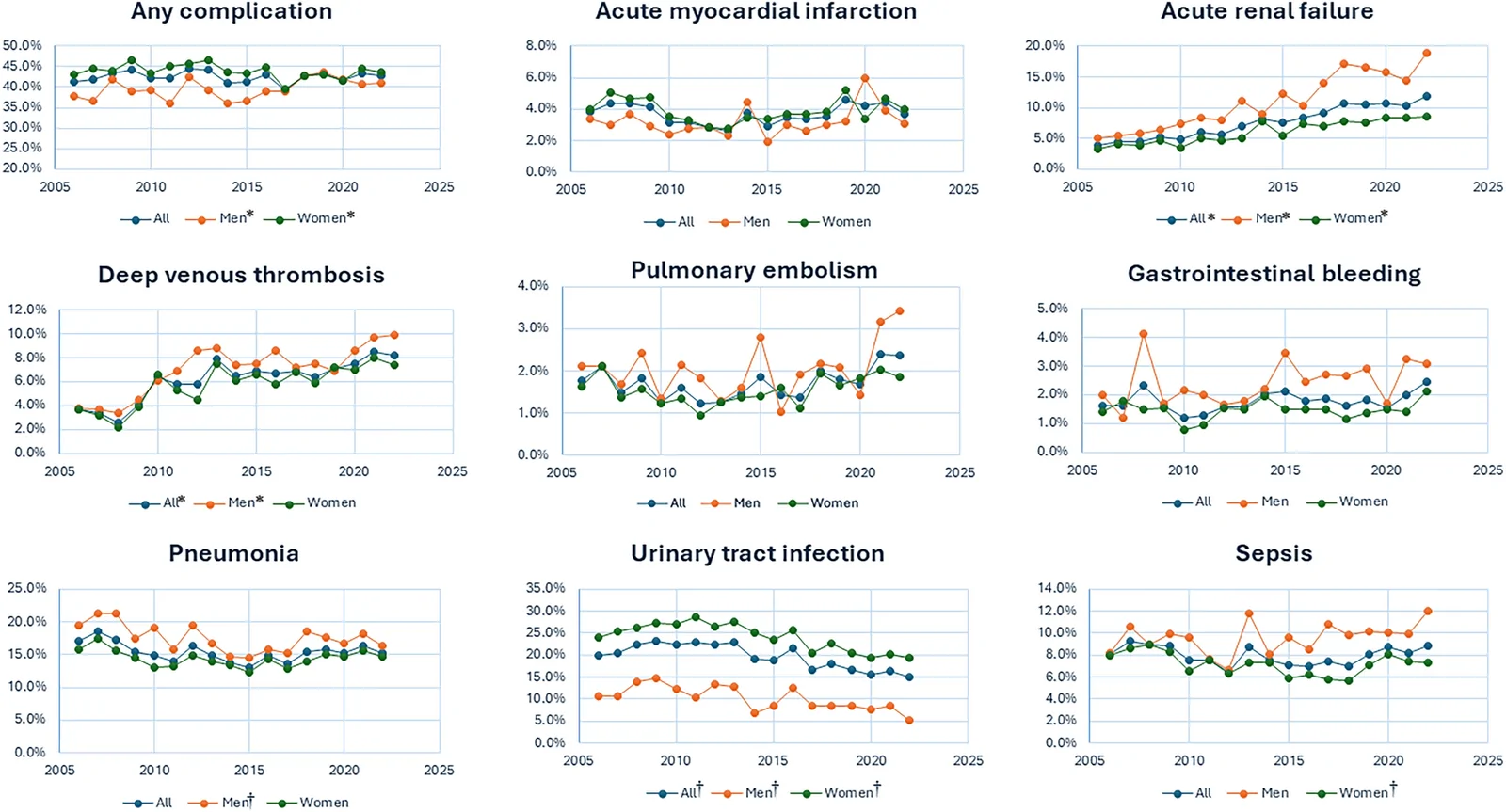
Age- and sex-adjusted trends in the prevalence of medical complications in aneurysmal subarachnoid hemorrhage in the USA from 2006 to 2022 according to sex. ^*^Indicates *p*-Value for trend toward increase over time < 0.05. ^†^Indicates *p*-Value for trend toward decline over time < 0.05. Absence of ^*^ or ^†^ indicates no significant change in trend over time

**Fig. 4:**
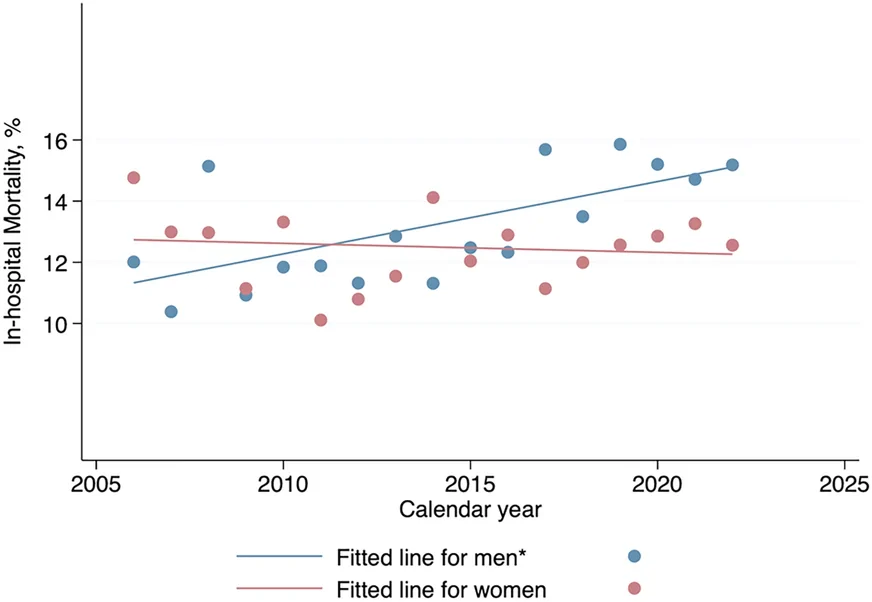
Trends in in-hospital mortality in aneurysmal subarachnoid hemorrhage in the USA from 2006 to 2022 according to sex. ^*^Represents *p*-value for trend < 0.05

The age- and sex-standardized prevalence of having at least one complication did not change over time (*p*-for-trend = 0.989). However, when stratified by sex, the age-standardized risk of having at least one complication increased marginally in male (*p*-trend = 0.041) but did not change over time in female (Fig. 3). The age- and sex-standardized prevalence of UTI declined over time in both sexes (all *p*-for-trends < 0.001), while that of pneumonia declined over time in male only (*p*-for-trend 0.001). However, this was counterbalanced by a near threefold increase in ARF and a greater than twofold increase in DVT prevalence over time. Dialysis utilization in patients with ARF across the study period was 7.2%, but this proportion declined over time (e-Fig. 5). Sepsis prevalence in female decreased over time (*p*-for-trend < 0.05), while AMI, GIB, and PE prevalence did not change over time (Fig. 3). Pneumonia prevalence did not change over time in female (*p*-for-trend = 0.368). Overall age- and sex-adjusted in-hospital mortality was 12.7%. There was a significant trend toward increase in unadjusted sex-stratified incidence over time in male (*p*-for-trend = 0.038) but not in female (*p*-for-trend = 0.065) (Fig. 4). However, in models that adjust for age, comorbidity disease burden and the NISSSS (eTable 4, model 4), there was a 2% decline (PRR 0.98, 95% CI: 0.97–1.00) in male and a 3% decline (PRR 0.97, 95% CI: 0.96–0.97) with each unit increase in year over time. This observed decline in in-hospital mortality is despite increased prevalence of do-not-resuscitate orders in the study population over time (eFig. 6).

### Multivariable Association of Selected Demographic and Clinical Factors with Complication Prevalence

After multivariable adjustment, female hospitalized with aSAH had an 8% higher prevalence of having at least one medical complication compared with male (PRR: 1.08, 95% CI: 1.05–1.11, *p* < 0.001), but this was driven mainly by a greater than twofold higher prevalence of UTI in female and a 16% higher prevalence of AMI in female vs. male because the prevalence of all other complications was higher in male compared with female (Table 1).

**Table 1:** Multivariable association of selected clinical hospitalization variables with medical complications in subarachnoid hemorrhage hospitalizations in the USA from 2006 to 2022

	Any complication			Sepsis			Pneumonia		
Variables	Prevalence risk ratio	95% CI	*p*-value	Prevalence risk ratio	95%CI	*p*-value	Prevalence risk ratio	95% CI	*p*-value
Female vs. male	1.08	1.05–1.11	< 0.001	0.75	0.62–0.81	< 0.001	0.82	0.78–0.86	< 0.001
Age in years									
40–59 vs. 18–39	1.13	1.08–1.18	< 0.001	0.98	0.86–1.11	0.727	1.20	1.10–1.32	< 0.001
60–79 vs. 18–39	1.29	1.22–1.36	< 0.001	1.01	0.87–1.17	0.893	1.30	1.18–1.44	< 0.001
≥ 80 vs. 18–39	1.33	1.23–1.42	< 0.001	1.00	0.79–1.27	0.999	1.31	1.13–1.53	0.001
Race and ethnicity									
Black vs. white	1.02	0.98–1.06	0.360	1.03	0.92–1.15	0.633	0.95	0.88–1.02	0.168
Hispanic vs. white	1.03	0.99–1.08	0.124	1.10	0.97–1.24	0.137	1.03	0.94–1.12	0.542
Asian/Pacific Islander vs. white	1.04	0.98–1.10	0.215	1.06	0.88–1.27	0.546	0.91	0.80–1.04	0.160
Other race and ethnicity vs. white	1.11	1.05–1.17	< 0.001	1.19	1.03–1.37	0.019	1.09	0.99–1.20	0.065
Any clipping vs. coiling	1.00	0.97–1.03	0.839	1.01	0.92–1.10	0.832	1.04	0.98–1.10	0.184
NISSSS, per unit increase	1.05	1.05–1.06	< 0.001	1.09	1.08–1.10	< 0.001	1.13	1.12–1.13	< 0.001
Elixhauser score, per unit increase	1.13	112–1.14	< 0.001	1.17	1.14–1.19	< 0.001	1.11	1.10–1.13	< 0.001
Year, per unit increase	0.98	0.97–0.98	< 0.001	0.96	0.95–0.97	< 0.001	0.96	0.95–0.97	< 0.001

	Urinary tract infection			Acute myocardial infarction			Acute renal failure		
	Prevalence risk ratio	95% CI	*p*-value	Prevalence risk ratio	95%CI	*p*-value	Prevalence risk ratio	95% CI	*p*-value
Female vs. male	2.29	2.15–2.44	< 0.001	1.16	1.02–1.32	0.020	0.51	0.47–0.55	< 0.001
Age in years									
40–59 vs. 18–39	1.18	1.08–1.29	< 0.001	2.05	1.55–2.70	< 0.001	1.05	0.91–1.21	0.483
60–79 vs. 18–39	1.45	1.32–1.59	< 0.001	2.54	1.89–3.42	< 0.001	1.38	1.18–1.62	< 0.001
≥ 80 vs. 18–39	1.49	1.31–1.69	< 0.001	2.82	1.94–4.09	< 0.001	2.20	1.80–2.68	< 0.001
Race and ethnicity									
Black vs. white	0.93	0.86–1.00	0.064	0.67	0.54–0.82	< 0.001	1.66	1.50–1.83	< 0.001
Hispanic vs. white	1.07	0.99–1.16	0.077	0.85	0.70–1.05	0.119	1.06	0.93–1.20	0.387
Asian/Pacific Islander vs. white	1.17	1.04–1.31	0.006	1.09	0.82–1.46	0.535	1.09	0.91–1.31	0.341
Other vs. white	1.07	0.98–1.17	0.154	1.19	0.94–1.49	0.151	1.16	1.03–1.31	0.016
Any clipping vs. coiling	0.99	0.94–1.05	0.780	0.79	0.68–0.93	0.005	1.00	0.92–1.09	0.992
NISSSS	1.02	1.01–1.02	< 0.001	1.09	1.08–1.10	< 0.001	1.06	1.05–1.07	< 0.001
Elixhauser score	1.11	1.10–1.13	< 0.001	1.15	1.11–1.18	< 0.001	1.27	1.25–1.29	< 0.001
Year	0.96	0.96–0.96	< 0.001	0.97	0.95–0.98	< 0.001	1.03	1.02–1.04	< 0.001

	Gastrointestinal bleeding			Deep venous thrombosis			Pulmonary embolism		
	Prevalence risk ratio	95% CI	*p*-value	Prevalence risk ratio	95%CI	*p*-value	Prevalence risk ratio	95% CI	*p*-value
Female vs. male	0.59	0.50–0.70	< 0.001	0.82	0.75–0.91	< 0.001	0.80	0.68–0.95	0.012
Age in years									
40–59 vs. 18–39	1.49	1.06–2.09	0.022	1.07	0.91–1.25	0.426	0.97	0.72–1.30	0.824
60–79 vs. 18–39	1.84	1.27–2.66	0.001	1.18	0.99–1.40	0.072	0.93	0.65–1.30	0.656
≥ 80 vs. 18–39	2.17	1.33–3.53	0.002	0.90	0.68–1.20	0.481	0.72	0.42–1.25	0.245
Race									
Black vs. white	1.17	0.92–1.49	0.208	1.28	1.13–1.45	< 0.001	1.07	0.85–1.35	0.570
Hispanic vs. white	1.07	0.81–1.41	0.625	1.08	1.07–1.08	< 0.001	0.81	0.61–1.08	0.145
Asians/Pacific Islander vs. white	1.20	0.79–1.82	0.390	0.97	0.77–1.22	0.794	0.31	0.15–0.64	0.002
Other vs. white	1.13	0.88–1.45	0.344	1.17	1.00–1.38	0.048	0.89	0.67–1.17	0.388
Any clipping vs. coiling	0.66	0.54–0.80	< 0.001	1.24	1.13–1.37	< 0.001	1.14	0.95–1.38	0.158
NISSSS	1.08	1.06–1.10	< 0.001	1.08	1.07–1.08	< 0.001	1.05	1.04–1.07	< 0.001
Elixhauser score	1.20	1.16–1.26	< 0.001	1.16	1.13–1.19	< 0.001	1.43	1.38–1.49	< 0.001
Year	0.97	0.95–1.00	0.032	1.03	1.01–1.04	< 0.001	0.96	0.94–0.99	0.003

This model was adjusted for age, sex, race, clipping vs. coiling, NISSSS, Elixhauser Comorbidity Score, insurance status, smoking status, hospital location/teaching status, and hospital region

*NISSSS* National Inpatient Sample Subarachnoid Hemorrhage Severity Score

Increasing comorbidity burden as measured by the Elixhauser Index was associated with increased prevalence of all complications. For example, each unit increase in Elixhauser score was associated with 17% higher risk of sepsis (PRR: 1.17, 95% CI: 1.14–1.19, *p* < 0.001). Clipping hospitalizations had a 34% lower prevalence of GIB (PRR: 0.66, 95% CI: 0.54–0.90, *p* < 0.001) and a 21% lower prevalence of AMI (PRR: 0.79, 95% CI: 0.68–0.93, *p* = 0.005), but 24% greater prevalence of DVT (PRR: 1.24, 95% CI: 1.13–1.37, *p* < 0.001) compared with coiling hospitalizations. Prevalence of all other complications did not differ by coiling vs. clipping status (Table 1).

In the fully adjusted models highlighted in Table 1, each unit increase in year was associated with a 2–4% reduction in the prevalence of all complications, except for ARF and DVT, where prevalence increased by 5% and 3%, respectively, per unit increase in year over the study period. Nested regression models demonstrated that in all complications, when adding stroke severity and comorbidity burden (Model 2 in eTable 3) to models that were adjusted for age and sex alone, the association of each unit increase in year with complication prevalence was attenuated toward the null or toward decline. This indicates that stroke severity and comorbidity disease burden explain some of the trends in these complications over time.

### Association Between Medical Complications and Poor Outcomes

All complications were associated with increased prevalence of poor outcomes, defined using the dichotomous NISSOM (Table 2). In mutually adjusted models where all complications were adjusted for their association with other complications, pneumonia and sepsis had the strongest association with poor outcome. Interestingly in these fully adjusted models, clipping was associated with 13% greater prevalence of poor outcome compared with coiling (PRR: 1.13, 95% CI: 1.10–1.15, *p* < 0.001).

**Table 2:** Association of medical complications with poor outcomes in aneurysmal subarachnoid hemorrhage hospitalizations in the USA from 2006 to 2022

Variable	Multivariate model 1			Multivariate model 2			Multivariate model 3		
	PR	95% CI	*p*-value	PR	95% CI	*p*-value	PR	95% CI	*p*-value
AMI	1.43	1.39–1.47	< 0.001	1.13	1.10–1.17	< 0.001	1.09	1.06–1.13	< 0.001
ARF	1.46	1.42–1.50	< 0.001	1.15	1.13–1.18	< 0.001	1.10	1.06–1.12	< 0.001
DVT	1.52	1.48–1.56	< 0.001	1.21	1.18–1.25	< 0.001	1.14	1.12–1.20	< 0.001
PE	1.53	1.46–1.60	< 0.001	1.18	1.12–1.24	< 0.001	1.06	1.07–1.11	0.027
GIB	1.46	1.39–1.52	< 0.001	1.18	1.12–1.23	< 0.001	1.11	1.06–1.16	< 0.001
Pneumonia	1.75	1.71–1.78	< 0.001	1.27	1.25–1.30	< 0.001	1.22	1.20–1.25	< 0.001
Sepsis	1.62	1.58–1.66	< 0.001	1.27	1.24–1.30	. < 0.001	1.17	1.14–1.20	< 0.001
UTI	1.25	1.22–1.28	< 0.001	1.15	1.13–1.18	< 0.001	1.13	1.1–1.16	< 0.001
Clipping vs. coiling	1.13	1.10–1.15	< 0.001	1.13	1.11–1.15	< 0.001	1.13	1.10–1.15	< 0.001

Multivariate model 1 was adjusted for age and sex; multivariate model 2 was adjusted for age, sex, race, clipping vs. coiling, NISSSS, Elixhauser Comorbidity Score, insurance status, smoking status, hospital location/teaching status, and hospital region; multivariate model 3 was a fully adjusted model, including all variables from models 1 and 2, in addition to exclusive medical complications

*NISSSS* National Inpatient Sample Subarachnoid Hemorrhage Severity Score

## Discussion

In this study, we provide national-level generalizable data on the prevalence of medical complications in aneurysmal subarachnoid hemorrhage hospitalizations in the USA from 2006 to 2022. These estimates could serve as quality benchmarks by which hospitals may nationally compare their complication burden. We highlight that the average age and comorbidity disease burden in aneurysmal subarachnoid hemorrhage hospitalizations increased over time. Approximately 40% of aSAH admissions in the USA over this period had at least one medical complication. Infectious complications such as pneumonia, UTI, and sepsis were the most prevalent, but ARF prevalence increased by more than 300% over time such that it was the most prevalent complication in male in 2022. All complications were positively associated with poor outcome after multivariable adjustment.

From a clinical perspective, the age- and sex-stratified estimates of complication burden highlighted in this study provide novel data on key demographic subgroups that may be particularly prone to complications and for which additional measures may be necessary to prevent them. For example, the prevalence of UTI in elderly female (greater than 30% in ≥ 80-year-olds and greater than 25% in 60–79-year-olds) is exceptionally high, while those of pneumonia (26.8%) and ARF (28.6%) in male ≥ 80 years are also worrisome. Prior studies have demonstrated that patients with aSAH are particularly prone to a wide range of infections, particularly pneumonia and UTIs, and these have been associated with prolonged hospital stays, delayed cerebral ischemia, and decreased functional recovery [14–16]. Factors such as immune dysregulation, neurogenic pulmonary edema, prolonged ICU stay, use of immunosuppressive medication, neurologic deficits, and mechanical ventilation after aSAH put patients at risk for these infections [17]. Bladder dysfunction and urethral catheterization further elevate the risk of UTIs, while dysphagia may increase aspiration pneumonia risk. Prior studies evaluating the prevalence of infectious complications after aSAH have yielded wide-ranging estimates from 5.2 to 29.7% for pneumonia and from 8.6 to 36.2% for UTI [3, 18, 19]. However, many of these studies were conducted prior to the considerable advancements in aSAH care that has occurred over the past decade, and these estimates may not be generalizable to the entire US population as they were mostly carried out at large academic centers [3], which treat the sickest aSAH patients. Our prevalence data highlight that nationally, these elderly patients may be most prone to these complications, and neurosurgical or critical care providers taking care of these patients may need to take additional measures, such as possible limiting urinary catheterization, to reduce infection risk.

Our study of trends over time highlights areas in the medical care of aSAH where some progress has been made nationally over time to reduce medical complication burden but also other new areas where additional efforts may be needed to curtail complication burden. In this study, prevalence of most infectious complications declined over time, indicating that current efforts to reduce nosocomial infections may be yielding significant gains in patients with aSAH [1, 20]. However, the increase in ARF and DVT prevalence indicates that these are complications where additional efforts are still needed. The reasons underlying the increased prevalence of ARF in this study cannot be elucidated in this retrospective analysis of an administrative database, but there are many potential contributory factors, such as an older and increasingly comorbid aSAH population, the heightened use of nephrotoxic drugs, and a growing dependence on contrast media for imaging and endovascular interventions [21, 22]. Both abrupt blood-pressure lowering and vasopresser-driven induced hypertension for vasospasm or delayed cerebral ischemia can lead to renal hypoperfusion and acute kidney injury (AKI) [23, 24]. The risk is amplified when high-sodium, hyperosmolar fluids (e.g., hypertonic saline) and osmotic/nephrotoxic agents, such as mannitol, are used, particularly in those with baseline renal disease, diabetes, or hypovolemia [25–27]. Current goal-directed therapy and Neurocritical Care Society (NCS) guidelines revolve around maintaining hydration with isotonic saline, aiming for euvolemia and avoiding hemodynamic instability or fluid overload, as these would worsen renal perfusion [28, 29]. In the current study, dialysis utilization in ARF hospitalizations declined over time, and this may be an indication that improved detection of mild cases of ARF over time may be partly contributory to the increased prevalence over time. This finding may be consistent with what was found in a national cohort of > 4 million hospitalizations in the Veterans Health Administration database over the period from 2008 to 2017, where ARF as diagnosed by serum creatinine criteria remained unchanged across the period, but ICD-9/ICD-10 coding for ARF, especially stages 1 and 2 (which will not indicate dialysis), increased over time [30]. A similar trend may also be occurring in patients with SAH. However, the strong association of ARF with mortality in this cohort indicates that these mild cases of ARF are clinically important.

Similarly, factors such as prolonged immobility, prolonged mechanical ventilation [31, 32], systemic inflammatory response, and post-aSAH prothrombotic state [33, 34] put patients with aSAH at risk of DVT, but reasons underlying the increasing trend in DVT over time are not very clear. Most certainly, factors such as increasing age and comorbidity disease burden of patients with aSAH may be potential explanatory factors; however, in models adjusted for age and comorbidity burden, DVT prevalence continued to increase over time, suggesting that additional factors are likely contributory. Improvement in clinical awareness and advances in imaging techniques over time may have led to increased DVT detection. Nonetheless, whether real, from increasing prevalence, or artifactual, from increased detection, the strong association of DVT with poor aSAH outcome indicates that current approaches to prevent DVT in patients with aSAH are suboptimal, and additional measures are needed to prevent continued increase in prevalence.

Consistent with prior studies showing better short-term outcomes with coiling as opposed to clipping [35, 36], we found a 13% greater prevalence of poor outcome with clipping vs. coiling. However, we noted some heterogeneity in prevalence of complications by treatment, with hospitalizations for clipping having a lower prevalence of AMI and GIB and a greater prevalence of DVT compared with coiling hospitalizations. These findings are somewhat unexpected and will need further investigation in prospectively collected data, as the reasons underlying these findings cannot be properly ascertained in this retrospective analysis of an administrative database. Cerebral vasospasm, elevated intracranial pressure, and low creatinine clearance are some previously described risk factors for GIB in aSAH [37]. Antiplatelet use in a select group of patients post-coiling or in those undergoing stent-assisted coiling may be associated with increased GIB risk following coiling, but additional explanation for increased AMI risk in this population warrants further study.

### Limitations and Strengths of Our Study

This study should be viewed within the context of its limitations. Although we relied on the HCUP-defined constellation of codes for most variables, we cannot exclude errors due to coding inaccuracies. Our study of trends relies on the implicit assumption that coding and clinical practices did not change over time, which was not the case. Our study crossed the period of transition from ICD-9 to ICD-10 coding in the USA, which may have led to some inconsistencies in complication reporting. Changes in coding and documentation practices during this period could have artifactually influenced observed trends. However, such misclassifications are likely random and will bias the size of effect estimates toward the null. It is also important to note that there might be significant overlap between sepsis, pneumonia, and UTI, as these are not likely coded independent of each other. Furthermore, there have been changes in hospital billing practices over time to incentivize the documentation of comorbidities and in-hospital complications, and such practices may lead to an artefactual increase in the burden of some complications over time. Moreover, the clinical definition for some complications, such as sepsis, changed over the study period. We report on the presence or absence of complications but are unable to show a temporal association between SAH and these complications. This study likely underestimates the overall burden of medical complications in aSAH as patients may continue to develop these complications following discharge from their primary aSAH hospitalization. Previous validation studies have indicated that ICD-10 codes are very specific for ARF, but they demonstrate low sensitivity, implying that the documented prevalence of ARF underrepresents the actual burden [38]. Consequently, our finding of increasing ARF prevalence over time likely represents a conservative estimate, and the true rise in AKI among patients with aSAH may be even greater than reported. Furthermore, because the NIS does not capture present-on-admission indicators, we could not distinguish ARF present on admission from ARF acquired during hospitalization. This inability to differentiate preexisting from iatrogenic ARF limits our ability to accurately assess the preventable fraction of ARF.

The findings of this study are only generalizable to aSAH hospitalizations in which aneurysmal clipping or coiling were performed and is not reflective of aSAH hospitalizations in which aneurysm securement was not performed. Patients with aSAH not undergoing clipping or coiling may have significantly different aSAH complication burden; however, we have relied on previously validated algorithms for patients with aSAH [8], which are restricted to those specifically undergoing securement. Although we adjusted for multiple comorbid clinical and hospitalization factors, the section of our results evaluating the association of complications or clipping vs. coiling with outcome should be viewed with caution, as there remains a strong potential for residual confounding by unmeasured or unstudied factors.

## Conclusions

Despite the increasing age and comorbidity disease burden in aneurysmal subarachnoid hemorrhage hospitalizations in the USA over the last two decades, the overall adjusted prevalence of medical complications in these hospitalizations did not change significantly over time. Infectious complications during aSAH hospitalizations declined, while ARF and DVT detection increased by more than twofold over time. All complications were associated with poor aSAH outcome, and additional efforts are needed to mitigate them.

## Supplementary Information

Below is the link to the electronic supplementary material.

Supplementary file1 (DOCX 40 KB)

Supplementary file2 (DOCX 12 KB)

Supplementary file3 (TIF 155 KB)

Supplementary file4 (TIF 166 KB)

Supplementary file5 (TIF 172 KB)

Supplementary file6 (TIF 146 KB)

## Abbreviations

aSAH: Aneurysmal subarachnoid hemorrhage
AKI: Acute kidney injury
AMI: Acute myocardial infarction
ARF: Acute renal failure
CCS: Clinical Classification Software
CI: Confidence interval
CKD: Chronic kidney disease
DCI: Delayed cerebral ischemia
DVT: Deep venous thrombosis
GIB: Gastrointestinal bleeding
HCUP: Healthcare Cost and Utilization Project
ICD: International Classification of Diseases
LOS: Length of stay
NIS: Nationwide Inpatient Sample
NIS-SOM: Nationwide Inpatient Sample Subarachnoid Hemorrhage Outcome Measure
NISSSS: Nationwide Inpatient Sample Subarachnoid Hemorrhage Severity Score
OR: Odds ratio
PE: Pulmonary embolism
PRR: Prevalence risk ratio
SAH: Subarachnoid hemorrhage
UTI: Urinary tract infection
