# Supplementary material for: Medical Complications After Aneurysmal Subarachnoid Hemorrhage: Analysis of Trends in US Admissions from 2006 to 2022
Source: Neurocrit Care. 2026 Feb 19;44(3):1025–35. doi: 10.1007/s12028-025-02443-6 (PMC13249634; doi:10.1007/s12028-025-02443-6)
Supplement: Supplementary file 1 — Supplementary file1 (DOCX 40 KB) [file 12028_2025_2443_MOESM1_ESM.docx]

**ONLINE DATA SUPPLEMENT**

**eTable 1. Coding definitions for covariates**

| Variable | ICD-9 codes | ICD-10 codes |
| --- | --- | --- |
| Coiling | ICD Procedural codes 3952, 3972, 3979 | ICD Procedural codes 03VG3BZ, 03VG3DZ, 03VG3HZ, 03VG3ZZ, 03VK3BZ, 03VK3DZ, 03VK3HZ, 03VK3ZZ, 03VL3BZ, 03VL3DZ, 03VL3HZ, 03VL3ZZ, 03VL3BZ, 03VL3DZ, 03VL3HZ, 03VL3ZZ, 03VP3BZ, 03VP3DZ, 03VP3HZ, 03VP3ZZ, 03VQ3BZ, 03VQ3DZ, 03VQ3HZ, 03VQ3ZZ, 03LG3BZ, 03LG3DZ, 03LG3ZZ, 03LL3BZ, 03LL3DZ, 03LL3ZZ, 03LK3BZ, 03LK3ZZ, 03LP3BZ, 03LP3DZ, 03LP3ZZ, 03LQ3BZ, 03LQ3DZ, 03LQ3ZZ |
| Clipping | ICD Procedural codes 3951 | ICD Procedural codes 03LG0CZ, 03LG0ZZ, 03VG0CZ, 03VL0CZ, 03VL0ZZ, 03VP0CZ, 03VP0ZZ, 03VQ0CZ, 03VQ0ZZ |
| Hydrocephalus | 3313, 3314 | G91xx, G94xx |
| Aphasia | 4381, 7843 | R47xx |
| Cranial nerve deficit | 37940, 37941, 37942, 37943, 37850, 37851, 37852, 37853, 37854, 37855, 37856 | G52xx , H490, H491, H492, H493, H494, H499, H570 |
| Hemiplegia/Hemiparesis | 4383, 4384, 4385 | G81xx-G83xx, I69xx |
| Coma/stupor | 78001, 78003, 78002, 78009 | R40xx |
| Mechanical ventilation | ICD-9 Procedural codes 967.x \|9604, 9605 | ICD-10 procedural codes 09HN8BZ, 0BH13EZ, 0BH17EZ, 0BH18EZ, 0CHY7BZ, 0CHY8BZ, 0DH57BZ, 0DH58BZ, 0WHQ73Z, 0WHQ7YZ, 5A1945Z, 5A1955Z |
| External ventricular drain | 022, 0221 | x'=="009000Z, 00900ZX, 00900ZZ, 009030Z, 00903ZZ, 00903ZX, 009040Z, 00904ZZ, 00904ZX" \|`x'=="009500Z, 00950ZZ, 00950ZX, 009530Z, 00953ZZ, 00953ZX, 009540Z, 00954ZX, 00954ZZ, 009100Z, 00910ZZ, 00910ZX, 009130Z, 00913ZX, 00913ZZ, 009140Z, 00914ZX, 00914ZZ, 00940ZZ, 009400Z, 00940ZX, 009430Z, 00943ZZ, 00943ZX, 009440Z, 00944ZZ, 00944ZX, 009600Z, 00960ZX, 00960ZZ, 009630Z, 00963ZX, 00963ZZ, 009640Z, 00964ZX, 00964ZZ, 009700Z, 00970ZZ, 00970ZX, 009730Z, 00973ZZ, 00973ZX, 009740Z, 00974ZZ, 00974ZX |
| Ventricular shunt | HCUP PR CCS 2 | HCUP PR CCS 2 |
| Tracheostomy | HCUP PR CCS 34 | HCUP PR CCS 34 |
| Percutaneous endoscopic gastrostomy | HCUP PR CCS 71 | HCUP PR CCS 71 |
| Acute myocardial infarction | HCUP CCS 100 | HCUP CCS 100 |
| Acute renal failure | HCUP CCS 157 | HCUP CCS 157 |
| Sepsis | HCUP CCS 2 | HCUP CCS 2 |
| Pneumonia | HCUP CCS 122 | HCUP CCS 122 |
| Gastrointestinal bleeding | HCUP CCS 153 | HCUP CCS 153 |
| Urinary tract infection | HCUP CCS 159 | HCUP CCS 159 |
| Deep venous thrombosis | 45341, 45342, 45340, 45381, 45382, 45383, 45384, 45385, 45386, 45387, 45389, 4539" | I8240, I82401, I82402, I82403, I82409, I82411, I82412, I8243, I82419, I82421, I82422, I82423, I82429, I82431, I82432, I82433, I82439 , I82441, I82442, I82443, I82449, I82451, I82452, I82453, I82459, I82461, I82462, I82463, I82469, I82491, I82492, I82493, I82499, I824Y, I824Y1, I824Y2, I824Y3, I824Y9, I824Z1, I824Z2, I824Z3, I824Z9, I8260, I82601, I82602, I82603, I82609, I82611, I82612, I82613, I82619, I82621, I82622, I82623, I82629, I82890, I8290 |
| Pulmonary embolism | 41511, 41512, 41513, 41519 | I260, I2601, I2602, I2609, I2690, I2692, I2693, I2694, I2699 |
| Dialysis | 3995,5498,3927,3942,3943, V4511, V4512, V562, V5631, V563, 5856, 40301, 40311, 40391, 40402, 40403, 40413, 40492, 40493, 4470, 45821, E8722, E8791, 7925, 7929, 99668, 99673 | 5A1D70Z, 5A1D80Z, 5A1D60Z, 3E1M39Z, Z491, Z492, Z493, Z490, Z992, Z9115, I120, I132, I1311, N185, N186, T81512, T81522, T81532, T81592, T8241, T8242, T8243, T8249, T85631, T85621, T85611, T85691, T8571 |

***HCUP CCS*** *stands for Healthcare Cost and Utilization Project Clinical Classification Software*

*ICD Stands for International Classification of Diseases*

**eTable 2 Age and/or sex-standardized prevalence of baseline characteristic variables in aneurysmal  subarachnoid hemorrhage admissions in the United States from 2006-2022 according to sex groups**

| **Variable** | **Total** | **Men** | **Women** | **P-value** |
| --- | --- | --- | --- | --- |
| Number, % | 33,202 | 31.8 | 68.2 | N/A |
| Weighted number, % | 163,349 | 31.8 | 68.2 | N/A |
|  |  |  |  |  |
| **Age, years, mean(SE)** | 55.6 | 53.2 | 56.8 | <0.001 |
| **Age in years, %** |  |  |  | <0.001 |
| 18-39 | 12.4 | 16.4 | 10.5 |  |
| 40-59 | 48.6 | 50.9 | 47.6 |  |
| 60-79 | 34.5 | 30.3 | 36.5 |  |
| >=80 years | 4.5 | 2.4 | 5.5 |  |
| **Race %** |  |  |  | <0.001 |
| Whites | 51.0 | 50.5 | 51.2 |  |
| Blacks | 15.2 | 13.6 | 15.9 |  |
| Hispanic | 13.0 | 14.9 | 12.2 |  |
| Asians/Pacific Islander | 4.4 | 4.0 | 4.6 |  |
| Other/unknown | 16.4 | 16.9 | 16.2 |  |
| **Insurance %** | |  |  | <0.001 |
| Medicare | 27.1 | 21.8 | 29.5 |  |
| Medicaid | 18.8 | 19.5 | 18.4 |  |
| Private insurance | 40.6 | 41.2 | 40.3 |  |
| Self pay | 9.0 | 11.2 | 7.9 |  |
| No charge | 0.9 | 1.1 | 0.7 |  |
| Other | 3.8 | 5.2 | 3.1 |  |
| **Hospital region %** | |  |  | <0.001 |
| Northeast | 17.6 | 17.7 | 17.6 |  |
| Midwest | 20.9 | 21.0 | 20.9 |  |
| South | 37.5 | 37.0 | 37.7 |  |
| West | 24.0 | 24.3 | 23.8 |  |
| **Hospital Location/Teaching status %** | | | | <0.001 |
| Rural | 0.6 | 0.7 | 0.5 |  |
| Urban Nonteaching | 7.5 | 7.2 | 7.6 |  |
| Teaching | 91.9 | 92.1 | 91.9 |  |
| **Clinical characteristics, %** |  |  |  |  |
| Hypertension | 65.6 | 65.4 | 65.6 | 0.7742 |
| Diabetes mellitus | 12.2 | 12.4 | 12.2 | 0.6505 |
| Dyslipidemia | 21.9 | 22.6 | 21.6 | <0.001 |
| Smoking | 14.8 | 17.0 | 13.8 | <0.001 |
| Coiling | 65.5 | 66.5 | 65.0 | 0.0110 |
| Any mechanical ventilation | 41.1 | 42.2 | 40.6 | 0.0092 |
| Mechanical ventilation within 24 hours of admission | 32.4 | 32.4 | 32.4 | 0.9263 |
| NISSSS, mean (SE) | 5.47 (0.06) | 5.52 (0.07) | 5.44(0.06) | 0.224 |
| Elixhauser comorbidity score Command mean (SE) | 3.15 (0.03) | 3.15 (0.03) | 3.15 (0.03) | 0.903 |

**NISSSS: National Inpatient Sample Subarachnoid Hemorrhage Severity Score*

**eTable 3: Adjusted Trends in aSAH Complications: Nested Regression Models Accounting for Age, Sex, Stroke Severity, and Comorbidity Burden**

| **Variables** | **Prevalence risk ratio** | **95%CI** | **P-value** | **Prevalence risk ratio** | **95%CI** | **P-value** |
| --- | --- | --- | --- | --- | --- | --- |
| **Any Complication** | | | | | | |
|  | **Model 1** | | | **Model 2** | | |
| Women | 1.06 | 1.03-1.09 | <0.001 | 1.08 | 1.05 - 1.11 | <0.001 |
| Age in years |  |  |  |  |  |  |
| 40-59 vs 18-39 | 1.13 | 1.08-1.18 | <0.001 | 1.11 | 1.06 - 1.17 | <0.001 |
| 60-79 vs 18-39 | 1.29 | 1.22-1.36 | <0.001 | 1.30 | 1.24 - 1.37 | <0.001 |
| >= 80 vs 18-39 | 1.33 | 1.23-1.42 | <0.001 | 1.37 | 1.28 - 1.47 | <0.001 |
| Year | 1.00 | 1.00-1.00 | 0.995 | 0.98 | 0.97 - 0.98 | <0.001 |
| Elixhauser scpre | N/A | N/A | N/A | 1.13 | 1.12 - 1.14 | <0.001 |
| NISSSS | N/A | N/A | N/A | 1.05 | 1.05 - 1.06 | <0.001 |
| **Acute myocardial infarction** | | | | | | |
| Women | 1.11 | 0.98 - 1.26 | 0.096 | 1.15 | 1.014 - 1.30 | 0.029 |
| Age in years |  |  |  |  |  |  |
| 40-59 vs 18-39 | 2.25 | 1.70 - 2.98 | <0.001 | 2.00 | 1.51 - 2.65 | <0.001 |
| 60-79 vs 18-39 | 3.39 | 2.55 - 4.50 | <0.001 | 2.63 | 1.99 - 3.50 | <0.001 |
| >= 80 vs 18-39 | 4.30 | 3.03 - 6.10 | <0.001 | 3.17 | 2.23 - 4.50 | <0.001 |
| Year | 1.00 | 0.99 - 1.20 | 0.666 | .98 | 0.96 - 0.99 | 0.003 |
| Elixhauser score | N/A | N/A | N/A | 1.14 | 1.11 - 1.17 | <0.001 |
| NISSSS | N/A | N/A | N/A | 1.09 | 1.08 - 1.10 | <0.001 |
| **Acute renal failure** | | | | | | |
| Women | 0.50 | 0.46 - 0.546 | <0.001 | 0.52 | 0.48 - 0.56 | <0.001 |
| Age in years |  |  |  |  |  |  |
| 40-59 vs 18-39 | 1.17 | 1.02 - 1.35 | <0.001 | 1.02 | 0.89 - 1.18 | 0.767 |
| 60-79 vs 18-39 | 1.90 | 1.63 - 2.19 | <0.001 | 1.40 | 1.21 - 1.61 | <0.001 |
| >= 80 vs 18-39 | 3.42 | 2.86 - 4.08 | <0.001 | 2.30 | 1.92 - 2.75 | <0.001 |
| Year | 1.07 | 1.06 - 1.08 | <0.001 | 1.04 | 1.03 - 1.05 | <0.001 |
| Elixhauser scpre | N/A | N/A | N/A | 1.27 | 1.24 - 1.29 | <0.001 |
| NISSSS | N/A | N/A | N/A | 1.06 | 1.06 - 1.07 | <0.001 |
| **Deep venous thrombosis** | | | | | | |
| Women | 0.80 | 0.74 - 0.89 | <0.001 | 0.84 | 0.76 - 0.92 | <0.001 |
| Age in years |  |  |  |  |  |  |
| 40-59 vs 18-39 | 1.17 | 1.00 - 1.37 | 0.050 | 1.05 | 0.90 - 1.23 | 0.529 |
| 60-79 vs 18-39 | 1.39 | 1.19 - 1.63 | <0.001 | 1.01 | 0.93 - 1.30 | 0.255 |
| >= 80 vs 18-39 | 1.06 | 0.81 - 1.38 | 0.671 | 0.79 | 0.60 - 1.03 | 0.084 |
| Year | 1.05 | 1.04 - 1.06 | <0.001 | 1.03 | 1.01 - 1.04 | <0.001 |
| Elixhauser score | N/A | N/A | N/A | 1.15 | 1.12 - 1.18 | <0.001 |
| NISSSS | N/A | N/A | N/A | 1.08 | 1.07 - 1.09 | <0.001 |
| **Pulmonary embolism** | | | | | | |
| Women | 0.77 | 0.65 - .92 | 0.003 | 0.81 | 0.69 - 0.97 | 0.018 |
| Age in years |  |  |  |  |  |  |
| 40-59 vs 18-39 | 1.17 | 0.87 - 1.57 | 0.289 | 0.96 | 0.72 - 1.29 | 0.812 |
| 60-79 vs 18-39 | 1.34 | 0.99 - 1.82 | 0.060 | 0.90 | 0.66 - 1.22 | 0.502 |
| >= 80 vs 18-39 | 1.17 | 0.72 - 1.91 | 0.533 | 0.67 | 0.41 - 1.10 | 0.110 |
| Year | 1.02 | 1.00 - 1.04 | 0.120 | 0.97 | 0.95 - 1.00 | 0.026 |
| Elixhauser score | N/A | N/A | N/A | 1.41 | 1.36 - 1.47 | <0.001 |
| NISSSS | N/A | N/A | N/A | 1.06 | 1.04 - 1.07 | <0.001 |
| GIB | | | | | | |
| Women | 0.56 | 0.48 - 0.67 | <0.001 | 0.59 | 0.50 - 0.69 | <0.001 |
| Age in years |  |  |  |  |  |  |
| 40-59 vs 18-39 | 1.67 | 1.19 - 2.34 | 0.003 | 1.46 | 1.04 - 2.06 | 0.027 |
| 60-79 vs 18-39 | 2.43 | 1.72 - 3.44 | <0.001 | 1.84 | 1.29 - 2.61 | 0.001 |
| >= 80 vs 18-39 | 3.30 | 2.14 - 5.09 | <0.001 | 2.32 | 1.49 - 3.61 | <0.001 |
| Year | 1.01 | 0.99 - 1.03 | 0.183 | 0.98 | 0.96 - 1.01 | 0.142 |
| Elixhauser score | N/A | N/A | N/A | 1.20 | 1.15 - 1.25 | <0.001 |
| NISSSS | N/A | N/A | N/A | 1.08 | 1.07 - 1.10 | <0.001 |
| **Urinary tract infection** | | | | | | |
| Women | 2.26 | 2.12 - 2.42 | <0.001 | 2.29 | 2.15 - 2.44 | <0.001 |
| Age in years |  |  |  |  |  |  |
| 40-59 vs 18-39 | 1.24 | 1.13 - 1.35 | <0.001 | 1.16 | 1.07 - 1.27 | 0.001 |
| 60-79 vs 18-39 | 1.66 | 1.52 - 1.81 | <0.001 | 1.47 | 1.35 - 1.61 | <0.001 |
| >= 80 vs 18-39 | 1.85 | 1.64 - 2.07 | <0.001 | 1.56 | 1.39 - 1.76 | <0.001 |
| Year | 0.98 | 0.97 - 0.98 | <0.001 | 0.97 | 0.96 - 0.97 | <0.001 |
| Elixhauser score | N/A | N/A | N/A | 1.10 | 1.09 - 1.12 | <0.001 |
| NISSSS | N/A | N/A | N/A | 1.02 | 1.01 - 1.02 | <0.001 |
| **Pneumonia** | | | | | | |
| Women | 0.79 | 0.75 - 0.84 | <0.001 | 0.82 | 0.78 - 0.87 | <0.001 |
| Age in years |  |  |  |  |  |  |
| 40-59 vs 18-39 | 1.32 | 1.20 - 1.46 | <0.001 | 1.18 | 1.07 - 1.29 | 0.001 |
| 60-79 vs 18-39 | 1.67 | 1.51 - 1.84 | <0.001 | 1.27 | 1.16 - 1.40 | <0.001 |
| >= 80 vs 18-39 | 1.75 | 1.51 - 2.04 | <0.001 | 1.29 | 1.12 - 1.49 | 0.001 |
| Year | 0.99 | 0.99 - 1.00 | 0.102 | 0.96 | 0.95 - 0.97 | <0.001 |
| Elixhauser | N/A | N/A |  | 1.11 | 1.10 - 1.13 | <0.001 |
| NISSSS | N/A | N/A |  | 1.13 | 1.12 - 1.13 | <0.001 |
| **Sepsis** | | | | | | |
| Women | 0.73 | 0.68 - 0.79 | <0.001 | 0.76 | 0.70 - 0.82 | <0.001 |
| Age in years |  |  |  |  |  |  |
| 40-59 vs 18-39 | 1.08 | 0.95 - 1.23 | 0.250 | 0.96 | 0.84 - 1.09 | 0.495 |
| 60-79 vs 18-39 | 1.36 | 1.20 - 1.56 | <0.001 | 1.05 | 0.92 - 1.19 | 0.492 |
| >= 80 vs 18-39 | 1.50 | 1.21 - 1.86 | <0.001 | 1.09 | 0.88 - 1.35 | 0.443 |
| Year | 0.99 | 0.99 - 1.01 | 0.497 | 0.97 | 0.96 - 0.98 | <0.001 |
| Elixhauser scpre | N/A | N/A | N/A | 1.16 | 1.13 - 1.18 | <0.001 |
| NISSSS | N/A | N/A | N/A | 1.09 | 1.08 - 1.10 | <0.001 |

Footnote:
*Multivariate Model 1 adjusted for age and sex. Multivariate Model 2 adjusted for age, sex, race, clipping vs. coiling, NISSSS, Elixhauser comorbidity score, insurance status, smoking status, hospital location/teaching status, and hospital region. Multivariate Model 3 was a fully adjusted model, including all variables from Models 1 and 2, in addition to exclusive medical complications*

Etable 4. Prevalence risk ratios for mortality in males vs females

| **Nested Model Variables** | **Male** | | | **Female** | | |
| --- | --- | --- | --- | --- | --- | --- |
|  | **PRR** | **95% CI** | **p-value** | **PRR** | **95% CI** | **p-value** |
| **Model 1** |  |  |  |  |  |  |
| Year | 1.02 | 1.01–1.03 | 0.003 | 1.00 | 0.99–1.00 | 0.625 |
| **Model 2** |  |  |  |  |  |  |
| Year | 1.01 | 1.00–1.03 | 0.033 | 0.99 | 0.98–1.00 | 0.037 |
| **Model 3** |  |  |  |  |  |  |
| Year | 1.00 | 0.99–1.02 | 0.370 | 0.99 | 0.98–1.00 | 0.011 |
| **Model 4** |  |  |  |  |  |  |
| Year | 0.98 | 0.97–1.00 | 0.009 | 0.97 | 0.96–0.97 | 0.000 |

*Model 1 adjusted for the year.*

*Model 2= Model 1 + age.*

*Model 3= Model 2 + the Elixhauser comorbidity score.*

*Model 4 = Model 3 + the National Inpatient Sample Subarachnoid Severity Score (NISSSS).*
