# Supplementary material for: Medical Complications After Aneurysmal Subarachnoid Hemorrhage: Analysis of Trends in US Admissions from 2006 to 2022
Source: Neurocrit Care. 2026 Feb 19;44(3):1025–35. doi: 10.1007/s12028-025-02443-6 (PMC13249634; doi:10.1007/s12028-025-02443-6)
Supplement: Supplementary file 2 — Supplementary file2 (DOCX 12 KB) [file 12028_2025_2443_MOESM2_ESM.docx]

eFigure 1. Trends in Age Distribution of Aneurysmal Sub-arachnoid Hemorrhage Patients 2006-2022. *P* value for trend <0.05 for all age groups

eFigure 2 Trends in Length of Stay (LOS) of Aneurysmal Sub-arachnoid Hemorrhage hospitalizations from 2006-2022.

eFigure 3 Trends in Mean Clinical Severity of SAH (NISSSS) score of Aneurysmal Sub-arachnoid Hemorrhage Patients from 2006-2022.

eFigure 4. Prevalence of medical complications in aneurysmal subarachnoid hemorrhage hospitalizations in the United States from 2006-2022 by age and sex.

eFigure 5. Prevalence of dialysis utilization from 2006-2022.

eFigure 6. Prevalence of Do-Not-Resuscitate orders in aneurysmal subarachnoid hemorrhage patients.
