# Supplementary figures and images for: Medical Complications After Aneurysmal Subarachnoid Hemorrhage: Analysis of Trends in US Admissions from 2006 to 2022
Source: Neurocrit Care. 2026 Feb 19;44(3):1025–35. doi: 10.1007/s12028-025-02443-6 (PMC13249634; doi:10.1007/s12028-025-02443-6)

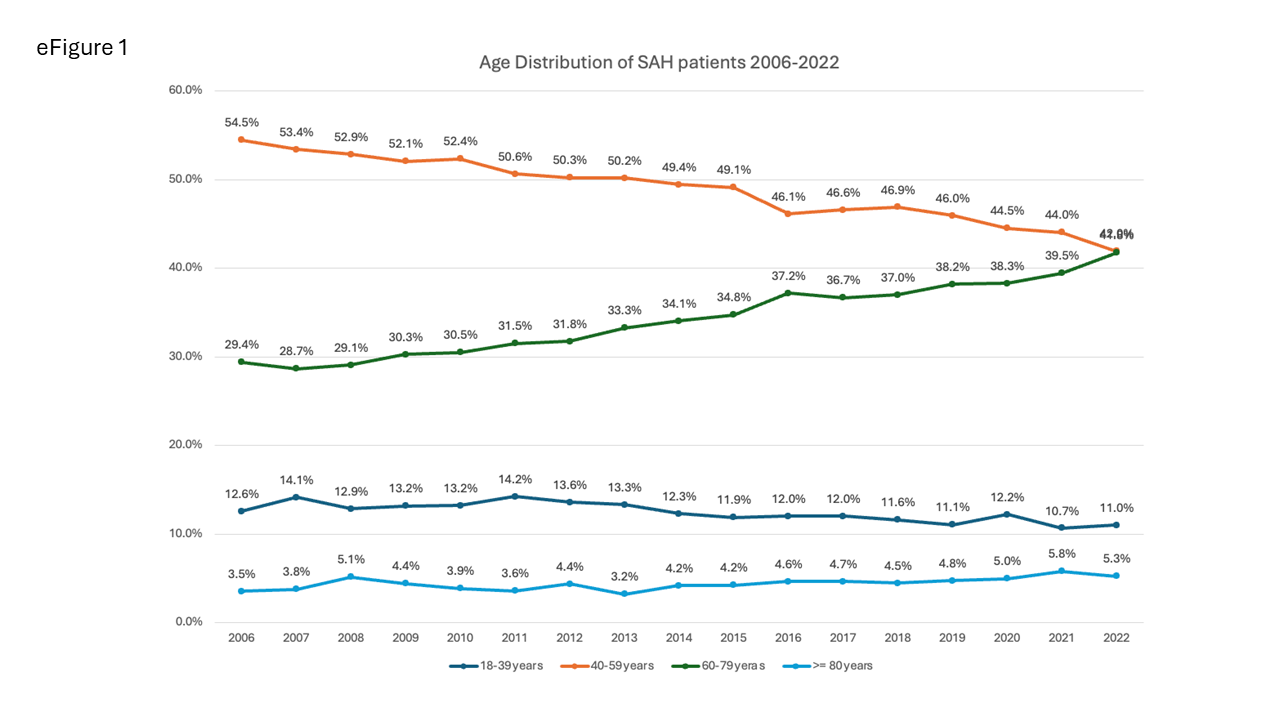

Supplement: Supplementary file 3 — Supplementary file3 (TIF 155 KB) [file 12028_2025_2443_MOESM3_ESM.tif]

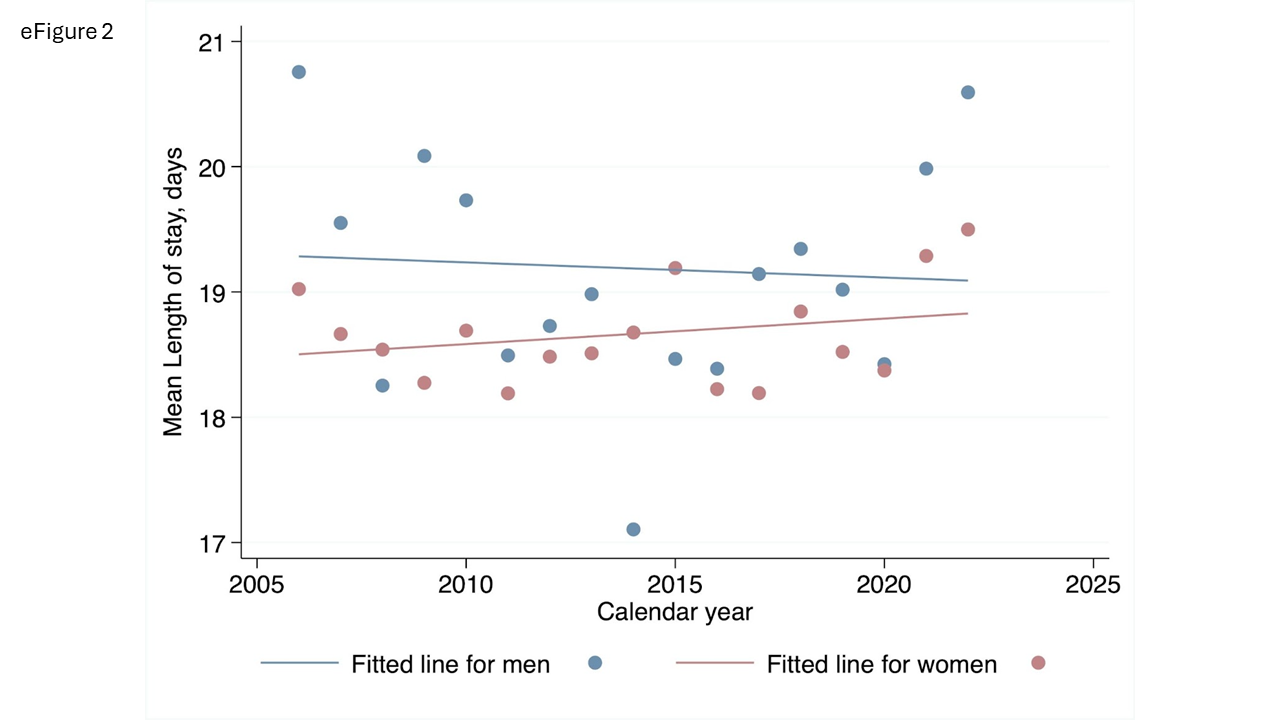

Supplement: Supplementary file 4 — Supplementary file4 (TIF 166 KB) [file 12028_2025_2443_MOESM4_ESM.tif]

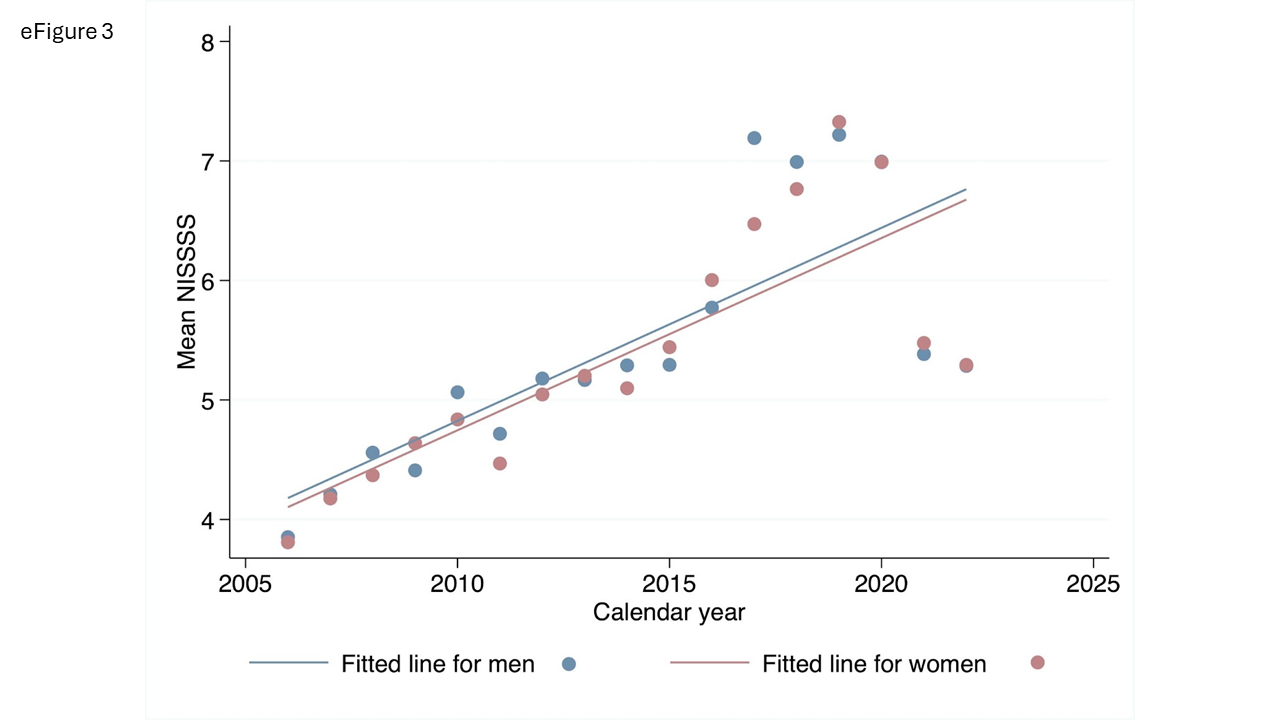

Supplement: Supplementary file 5 — Supplementary file5 (TIF 172 KB) [file 12028_2025_2443_MOESM5_ESM.tif]

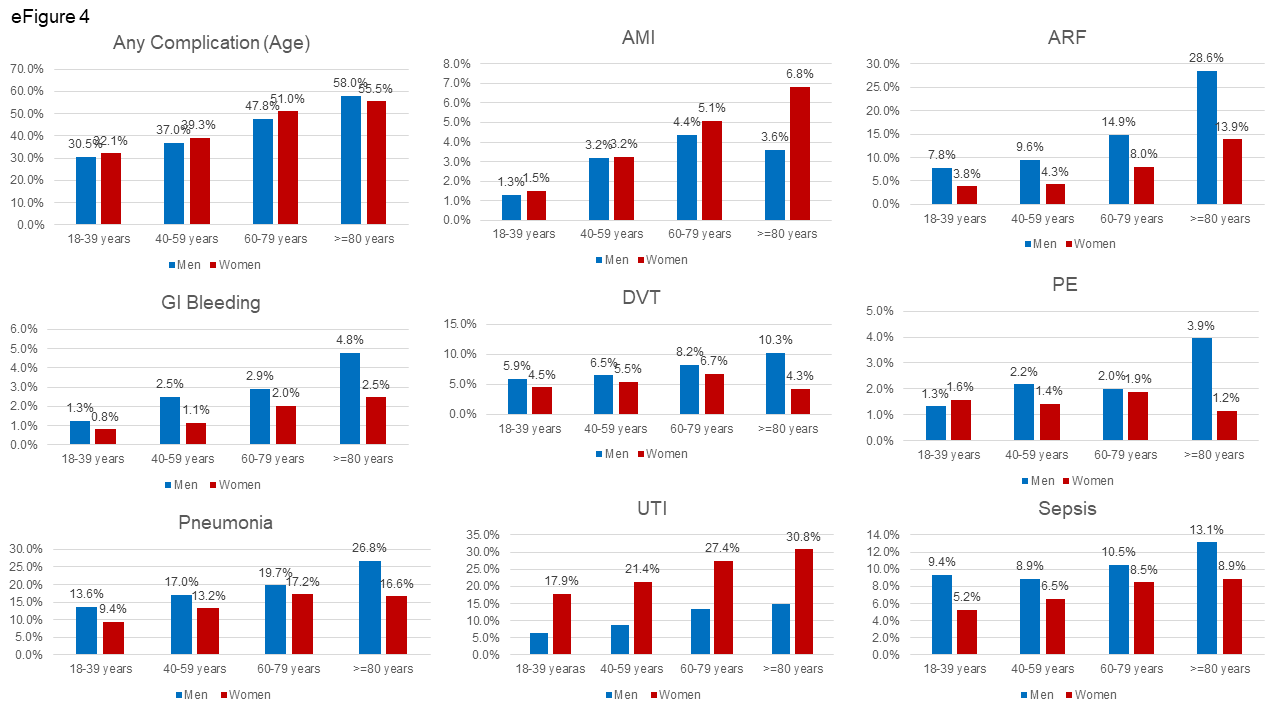

Supplement: Supplementary file 6 — Supplementary file6 (TIF 146 KB) [file 12028_2025_2443_MOESM6_ESM.tif]
